# Assessment of early and post COVID-19 vaccination antibody response in healthcare workers: a critical review

**DOI:** 10.1017/S0950268823000390

**Published:** 2023-03-20

**Authors:** Brandon Goodwin, Hasan Zia, David F. Lo

**Affiliations:** 1 Department of Medicine, Rowan University School of Osteopathic Medicine, Stratford, NJ, USA; 2 American Preventive Screening & Education Association (APSEA), Stratford, NJ, USA; 3 Department of Biology, Rutgers, The State University of New Jersey, New Brunswick, NJ, USA

We read with great interest the article by Mansour *et al*. titled, ‘*Assessment of Early and Post COVID-19 Vaccination Antibody Response in Healthcare Workers: A Multicenter Cross-Sectional Study on Inactivated, mRNA, and Vector-Based Vaccines*’ and would like to offer additional insight and feedback on their fine work [1]. We hope that our feedback is valuable in furthering the conversation and scientific inquiry into the understanding of COVID vaccine-induced humoral immunity.

Mansour *et al*. summarise that healthcare workers (HCWs) who had prior exposure to COVID-19 had more significant response rates to vaccination than those who did not [1]. In their study, the researchers discerned that a higher body mass index was statistically correlated with higher levels of serum antibody levels following the second vaccination. The study was a prospective, multi-centre, cross-sectional study and conducted in three major hospitals in Iran. The study included 962 HCWs, with 663 female and 299 male respondents. Participants had their blood drawn, and then spun and assayed to determine the number of antibodies. Following the ELISA testing, statistical analysis was conducted to determine the degree of significance.

First, it is important to operationally define the researchers' population of interest. For instance, the authors state that they are investigating antibody load in healthcare professionals (HCPs), but often continue to use the term interchangeably with HCWs. These terms are not synonymous, and their use in this paper is not mutually exclusive. This lack of operational definition leads to potential confusion when reading the paper. Furthermore, the researchers included HCWs who had any possible exposure to COVID-contaminated products or material in their analysis. These individuals are less likely to contract COVID-19, as fomite spread is considered highly unlikely in real-life scenarios [2]. As such, this potentially confounds their desired outcome of HCPs' antibody levels when HCWs have a significantly lower risk of infection.

Moreover, it is important to note that out of the 962 HCWs included in this study, only 33 had a positive history of infection (3.4%). The authors do not delineate if this statistic is from self-report or from previous testing of antibody titres. The researchers note that COVID-19 infections can often be silent or asymptomatic. As such, it is imperative to note that some of the respondents may have had prior infection without their knowledge and had not reported it, confounding the results of this study. This study ran from 2020 to 2021, and it is surprising to see that only 3.4% of participants contracted a disease, which saw 7.5 million individuals infected with COVID-19 in a country of 88 million, or an 8.5% national infection rate (https://COVID19.who.int/region/emro/country/ir). It stands to reason that HCPs who are actively seeing sick patients who may/may not have Coronavirus should have a higher infectivity rate than the national average.

Finally, the researchers note that research participants who had prior infection with COVID-19 had a higher antibody titre once vaccinated. This is not a novel concept, but exactly how humoral immunity works. The core tenet of humoral immunity is that the more the body is exposed to an antigen, the earlier and more significant the immune response. It is no surprise that the researchers discerned this with their statistical analysis.

Ultimately, the study is subject to self-report bias and operational definitions which were not clearly defined. We applaud the work of Mansour *et al*. for an interesting and unique study and hope that our feedback helps further the conversation on COVID vaccinations and antigen levels in HCPs/workers.
